# The impact of daytime transoral neuromuscular stimulation on upper airway physiology – A mechanistic clinical investigation

**DOI:** 10.14814/phy2.15360

**Published:** 2022-06-24

**Authors:** Brandon Nokes, Christopher N. Schmickl, Rebbecca Brena, Nana Naa‐Oye Bosompra, Dillon Gilbertson, Scott A. Sands, Rakesh Bhattacharjee, Dwayne L. Mann, Robert L. Owens, Atul Malhotra, Jeremy E. Orr

**Affiliations:** ^1^ University of California Division of Pulmonary, Critical Care, Sleep Medicine, and Physiology La Jolla California USA; ^2^ VA San Diego Division of Sleep Medicine San Diego California USA; ^3^ Division of Sleep and Circadian Disorders Brigham and Women's Hospital and Harvard Medical School Boston Massachusetts USA; ^4^ Rady's Children's Hospital San Diego California USA; ^5^ Institute for Social Science Research University of Queensland Brisbane Australia

## Abstract

There is a need for alternatives to positive airway pressure for the treatment of obstructive sleep apnea and snoring. Improving upper airway dilator function might alleviate upper airway obstruction. We hypothesized that transoral neuromuscular stimulation would reduce upper airway collapse in concert with improvement in genioglossal muscle function. Subjects with simple snoring and mild OSA (AHI < 15/h on screening) underwent in‐laboratory polysomnography with concurrent genioglossal electromyography (EMGgg) before and after 4–6 weeks of twice‐daily transoral neuromuscular stimulation. Twenty patients completed the study: Sixteen males, mean ± SD age 40 ± 13 years, and BMI 26.3 ± 3.8 kg/m^2^. Although there was no change in non‐rapid eye movement EMGgg phasic (*p* = 0.66) or tonic activity (*p* = 0.83), and no decrease in snoring or flow limitation, treatment was associated with improvements in tongue endurance, sleep quality, and sleep efficiency. In this protocol, transoral neurostimulation did not result in changes in genioglossal activity or upper airway collapse, but other beneficial effects were noted suggesting a need for additional mechanistic investigation.

## Abstract 2

Daytime transoral neuromuscular stimulation is a novel treatment for mild obstructive sleep apnea and snoring. We sought to evaluate the mechanism by which this method of sleep apnea improves disease severity. There was no change in neural drive to genioglossus, but there was an improvement in tongue endurance.

KEYWORDSflow‐limited breathing,genioglossus,obstructive sleep apneasnoring,upper airway training,

## 1 INTRODUCTION

Upper airway collapse during sleep is a prevalent phenomenon with both anatomical and functional determinants (Schmickl et al., in press). Simple snoring is a less severe variant of upper airway collapse characterized by stable flow‐limited breathing (Counter & Wilson, 2004). Despite the reduced severity of flow limitation, the medical and social impact of snoring is substantial (Counter & Wilson, 2004), yet often therapies such as positive airway pressure (PAP) or other procedures are not available for so‐called “simple snoring.” Those with more severe upper airway collapse and corresponding instability in breathing develop obstructive sleep apnea (OSA), with a large range in underlying physiology and corresponding OSA severity. Recent estimates suggest that OSA affects up to 1 billion people worldwide (Benjafield et al., 2019). Nasal CPAP is generally considered first line treatment and has clear benefits for some patients, but adherence is poor among others (Weaver et al., 1997; Weaver et al., 2007; Weaver et al., 2003). Alternative therapies such as oral appliances and upper airway surgery have variable efficacy with little ability to predict who might be responsive. With these considerations in mind, new therapies for flow‐limited breathing would be welcome.

Recent evidence suggests that OSA is multifactorial with mechanisms underlying OSA being highly variable across individuals (Schmickl et al., in press; Jordan et al., 2014). Beyond a collapsible upper airway, a sizable subset of OSA patients have abnormalities in upper airway muscle function and may be amenable to muscle training (Saboisky et al., 2007; Saboisky et al., 2009; Saboisky et al., 2012; Saboisky et al., 2014). For example, we have previously reported that OSA patients have increased tongue strength but reduced muscle endurance as compared to controls, potentially reflecting a role for tongue endurance training (Eckert et al., 2011). Although findings are variable, upper airway muscle training has also been successfully employed for simple snoring (Goswami et al., 2019). This training can include tongue and vocal exercises, as well as playing the didgeridoo (Puhan et al., 2006).

Direct stimulation of upper airway muscles has been examined with evidence for some benefits. Implantable hypoglossal nerve stimulators are used to activate the genioglossus directly during sleep to dilate the airway, but resultant changes in upper airway dilator activity and underlying muscle fiber properties suggest a training effect (Schwartz et al., 1985; Zaidi et al., 2013). Of note hypoglossal nerve stimulation is not FDA‐approved for those with snoring or mild OSA regardless of symptoms, and predicting therapeutic response remains a challenge (Strohl et al., 2017). Additional non‐invasive therapies focused on stimulating upper airway dilators may be an attractive target for those with snoring and mild OSA, particularly given limited therapeutic options for this group.

With this background, we sought to test the hypothesis that transoral neuromuscular electrical stimulation (NMES) of the tongue during wakefulness would lead to improvements in upper airway muscle physiology during sleep, as assessed by genioglossal electromyography (EMGgg) and upper airway obstruction for individuals in the spectrum of simple snoring to mild OSA.

## 2 METHODS

All patients signed informed consent. Final study design was approved by the University of California San Diego (UCSD) Human Research Protections Program (HRPP#181359). All study visits took place at UCSD from July to December 2019.

### 2.1 Inclusion criteria

We recruited men and women who were 18–65 years of age. We required confirmation of snoring by a live‐in bed partner reporting ≥6 months history of habitual snoring (i.e., > 5 days per week).

### 2.2 Exclusion criteria

We excluded patients with a pacemaker or other implanted medical electrical devices, current or recent (within last 6 months) treatment for snoring or sleep apnea, previous oral or pharyngeal surgery other than dental procedures, those with craniofacial skeletal or muscular abnormalities, a history of driving or other accidents due to sleepiness or an Epworth sleepiness score (ESS) > 18/24. Pregnant subjects were excluded as well as individuals with known cardiac (other than hypertension), pulmonary, renal, neurologic, neuromuscular, hepatic disease or psychiatric disorders (other than depression or anxiety). Other exclusion criteria included BMI > 35 kg/m^2^ (due to the increased likelihood of increased baseline AHI), non‐English speakers (due to necessity to complete validated questionnaires), inability to complete daily neuromuscular stimulation, presence of other sleep disorders, presence of tongue or lip piercing. Subjects could not be on medications with sedative or myorelaxant properties or effects on cardiac or pulmonary function. They were also excluded if they had substantial alcohol use (>3 oz/day), use of illicit drugs. We excluded subjects if their AHI was >15/h on screening home sleep test or snoring less than 20% of total sleep time during baseline polysomnography.

### 2.3 Screening

Prospective subjects were recruited from the general population via social media and from sleep clinics via flyers. Initial screening by phone was followed by in‐person screening and written informed consent was then obtained. Participants lacking recent (<6 months) sleep study results underwent screening using ApneaLink (ResMed) home testing. Subjects were excluded if their AHI was >15/h.

### 2.4 Baseline visit

Participants underwent physical assessment (height, weight, neck and waist circumference, and blood pressure) and completed questionnaires to assess sleep quality and sleepiness (Pittsburgh Sleep Quality Index (PSQI) and Epworth Sleepiness Scale (ESS)) for the prior month. Participants were then instructed to use the Iowa oral performance instrument (IOPI) to determine tongue protrusion force and fatigability. This test involved participants' squeezing a disposable plastic bulb with their tongue against the roof of the mouth for several seconds to establish a maximum effort. Then, endurance was determined as the duration for which half the maximum pressure could be maintained. This process was repeated three times with 60 seconds of rest between each trial. Tongue protrusion strength was determined as the highest pressure achieved from three attempts and endurance was determined as the duration for which half the maximum pressure could be maintained (Adams et al., 2013). To assess objective sleepiness, participants underwent a 10‐minute psychomotor vigilance test (PVT) on a computer which required striking a key on the keyboard as quickly as possible following a randomly timed stimulus on the screen. These tasks were performed just prior to in‐lab polysomnogram on both visits.

### 2.5 Research polysomnography

Polysomnography was performed using a Natus Grass amplifier and CED 1401 analog to digital converter recording into Spike2 software. Electrodes were placed to record electroencephalogram (EEG), electrooculogram (EOG), electrocardiogram (EKG), chin electromyogram (EMG), and leg movements. Respiratory inductance elasticized bands were placed around the abdomen and chest to detect respiratory motion. A mask and calibrated pneumotachometer recorded air flow. Blood oxyhemoglobin saturation was monitored by finger probe. Body position, monitored by video, was restricted to the supine position to avoid postural effects on upper airway muscle control and AHI.

To measure genioglossal muscle activity (EMGgg) the floor of the mouth was topically anesthetized by placing a lidocaine‐soaked cotton swab (containing ~4 ml of 4% lidocaine) under the tongue. Two 25‐gauge needles each containing a Teflon‐coated stainless‐steel recording wire (<1 mm in diameter with ~1 mm at the tip bared of Teflon and bent to form a small hook) were placed perorally 1.5–2 cm into the right and left sides of the body of the genioglossus muscle. The needles were inserted perpendicular to the oral mucosa 3–4 mm laterally to the frenulum and posterior to the salivary duct. Immediately after insertion, the needles were removed leaving the small recording wires in place. After the genioglossal electromyogram electrodes were secured, muscle activity was recorded while participants performed quiet wakeful breathing before being instructed to swallow and “sniff” (inhale sharply through the nose). Subjects then repeated the tongue protrusion force and fatigue maneuvers.

Then the lights were turned off and participants were allowed to go to sleep in a dark, sound‐proof room at approximately 10:30 pm and were woken at 6 am.

### 2.6 Muscle training treatment

In the morning after polysomnography, participants were given the ExciteOSA transoral neurostimulation device (Signifier Medical Technologies) and were instructed on proper use. Using the silicone mouthpiece, participants placed the probes under the tongue and followed the instructions on the phone‐based application. The device provided gentle electrical stimulation as titrated by the participant, causing stimulation of the tongue. Participants took the device home and were asked to use it for 20 minutes in the morning and at night every day for 4–6 weeks. The device recorded usage time to allow assessment of adherence. Additionally, on the 7th day each week, participants were contacted to assess for any potential issues.

### 2.7 Device settings

The participants were instructed to self‐titrate the device based on maximum tolerability without discomfort. The voltage delivered by the device is tailored towards the patient's tongue resistance. The patient has complete control of the intensity of therapy which ranges from 1–15. Depending on the tongue resistance of the patient (calculated by the induction current at the start of the therapy), the patient will receive any way between 1.35v at level 1 to 29 V at level 15.

### 2.8 Follow up visit

Subjects returned to the research laboratory for a follow up visit after 4–6 weeks of treatment. Research procedures were identical to those performed on the baseline visit. Adherence was defined as the number of sessions in which the device was used as intended (i.e., percent of 20‐min sessions completed).

#### 2.8.1 Power analysis

Based on feasibility, we aimed for 20 subjects to complete the protocol. Using a paired *t*‐test this sample size provided power of 80% with a two‐tailed alpha of 0.05 to detect an effect size of 0.66 units (Cohen's d) for change in genioglossal activity between baseline and follow up.

#### 2.8.2 Signal processing

Sleep and ventilation were scored from de‐identified studies by a single experienced registered polysomnographic technologist (RPSGT) blinded to treatment (baseline/follow‐up). Scoring and initial processing were performed in Spike2. Apneas and hypopneas were defined using AASM criteria, defined as, a ≥ 30% reduction in airflow with cortical arousal or a ≥ 3% oxygen desaturation. (Berry et al., 2017). Snoring was quantified by audio signals by our sleep technologists as the time spent snoring/total NREM sleep time as recorded by an in‐lab microphone (Arnardottir et al., 2016). Arousals were scored in accordance with AASM guidelines, as a sudden shift in EEG for 3 s or greater in NREM, while in REM a sudden shift in EEG accompanied by an increase in chin tone for 1 s or greater.

EMGgg signals were amplified and band‐pass filtered (30 Hz–1 KHz). Raw signals were processed offline in Spike2 using DC removal (0.7 second time constant), then rectified and smoothed (0.1 second time constant). We then quantified this transformed signal as a percent of the best available maximum maneuver (tongue protrusion, sniff, or swallow) with the highest single value being our maximum value (100%) in reference to electrical zero (0%). For each individual, the type of reference maximum maneuver used (tongue protrusion, sniff, or swallow) was kept consistent from baseline to follow up, but the value used for each night was from that night's recording.

Subsequent signal analysis was performed in Matlab (Mathworks Inc.) using established scripts (Mann et al., 2019). Tonic and phasic genioglossus activity (as %Max) was determined on a breath‐by‐breath basis over the night and recorded alongside sleep stage to determine median values for wake, NREM, REM, and all sleep. Breaths having any scored arousals were excluded. The severity of flow limitation for each breath was quantified via a previously validated method using parameters determined from analysis of the shape of each breath, reported as the ratio of observed flow to respiratory drive (“flow: drive”; lower values correspond with more severe flow limitation) (Mann et al., 2019). Similar to EMGgg, breath‐by‐breath flow limitation was recorded alongside sleep stage to determine median values for wake, NREM, REM and all sleep. Breaths including scored arousals were excluded.

#### 2.8.3 Statistical analysis

Data were analyzed using SPSS version 26 (IBM *SPSS* Statistics, IBM Corporation) and statistical software R 4.0.2. (www.rproject.org). Prespecified primary outcome measures were the change in genioglossal activity during NREM and percent sleep time snoring. Given the difficulty in quantifying snoring and recently published validation in quantification of flow limited breathing, we considered the flow: Drive score as an additional measure of inspiratory flow limitation alongside snoring. Secondary outcomes analyzed were considered exploratory. Data were analyzed using student's paired *t* tests or the Wilcoxon signed‐rank test for non‐normally distributed variables. In the case of missing data, sensitivity analysis was performed to compare pre and post means using mixed linear modeling.

#### 2.8.4 Clinical trials registration

The study was registered on Clinicaltrials.gov (NCT03913494).

## 3 RESULTS

**FIGURE 1:**
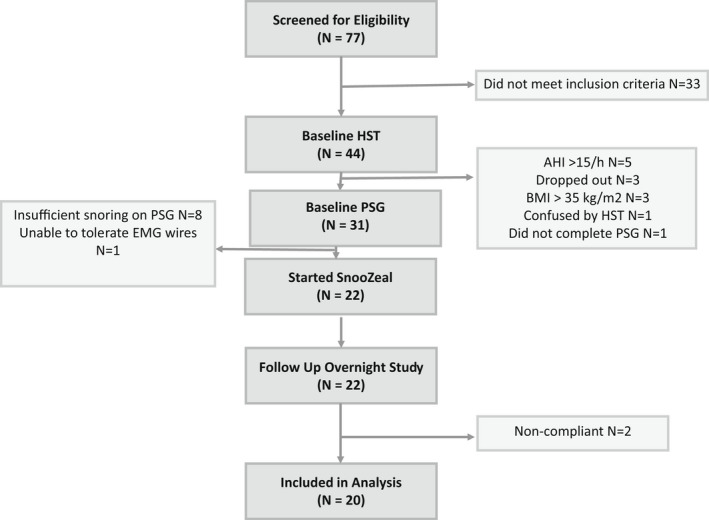
Enrollment flowchart.

Seventy‐seven subjects were screened with forty‐four completing baseline home sleep tests. Eventually 22 subjects were enrolled, of which two were not adherent with the device and thus excluded from the analysis (Figure 1). Among the 20 subjects finally included, the mean age was 40 ± 13 years with a BMI of 26.3 ± 3.8 kg/m^2^ and a mean home sleep test AHI of 5.9 ± 3.9 events/h. Sixteen of twenty subjects were male.

Each participant used the device during the period ranging from 28–45 days (mean 35 days ± SD 3.8). Data from 1124 sessions of device usage were collected. The mean number of sessions per user was 54 ± 12.1, ranging from 21 to 71. The percentage of days with at least one session was 86%+/−16.8%, ranging from 33% to 100%. The median percentage of days with two completed sessions was 67 ± 21.4%, ranging from 25% to 100%.

### 3.1 Primary outcomes

Sixteen subjects had EMGgg data available on both nights; four subjects had a single night in which EMGgg signals were not usable, either due to excess impedance or wire dislocation. As shown in Table 1 and Figure 1a and b, there was no significant difference in mean phasic EMGgg or mean tonic EMGgg during NREM sleep. Similar results were seen when examining all sleep (NREM + REM).

**TABLE 1:** Genioglossus EMG and flow limitation measures at baseline and follow‐up visit (* indicates significant *p*‐value)

	Pre‐treatment	Post‐treatment	Mean difference (95% CI)	*p*‐value
	Mean or median SD or [IQR]	Mean or median (SD) or [IQR]		
GG Phasic NREM (% max)	14.3 (16.5)	17.1 (18.5)	+2.8 (−10.3 to 15.9)	0.658
GG Tonic NREM (% max)	6.0 (6.1)	6.4 (6.6)	+0.5 (−3.9 to 4.9)	0.827
GG Phasic all sleep (% max)	14.0 (16.3)	15.7 (17.8)	+1.7 (−11.1 to 14.6)	0.776
GG Tonic all sleep (% max)	5.2 (5.0)	5.8 (6.0)	+0.6 (−3.3 to 4.4)	0.757
Time snoring (% NREM)	47 [28 to 63]	45 [21–72]	−2 (36.1 to 57.1)	0.54
Flow: drive NREM %	0.73 (0.12)	0.74 (0.10)	+0.01 (−0.05 to 0.07)	0.706
Flow:drive all sleep %	0.73 (0.12)	0.74 (0.10)	+0.01 (−0.05 to 0.05)	0.762
Sleep Efficiency, %	75 (11)	84 (10)	+12 (4.7 to 17.2)	0.002*
Duration Sleep NREM (minutes)	298 [267 to 338]	291 [248–323]	−7 (−36.2 to 21.1)	0.58
Duration sleep REM (minutes)	66 [33 to 83]	54 [30–64]	−12 (−50 to 1.3)	0.06
AHI, h^−1^	6 [2 to 15]	6 [1–18]	0 (−8.8 to 5.5)	0.67
AHI NREM, h^−1^	4 [2 to 14]	6 [0–19]	+2 (−6.0 to 7.9)	0.24
AHI REM, h^−1^	7 [0 to 14]	4 (1–8)	−3 (8.7 to 3.0)	0.32
Total Arousal index, h^−1^	21 (14)	19 (14)	−2 (−8.2 to 4.8)	0.58
Nadir oxygen saturation (%)	86 (4)	86 (5)	+1 (−2.1 to 1.0)	0.46
Mean nocturnal saturation (%)	94 (1)	94 (1)	0 (−0.9 to 0.07)	0.09

Polysomnographic measures at baseline and follow‐up visit. Sleep efficiency was calculated as total sleep time/total time in bed. Mean ± (SD) is reported, unless there was non‐normal distribution, median and interquartile range [IQR] are reported. *p*‐values are from paired *t*‐tests.

**FIGURE 2:**
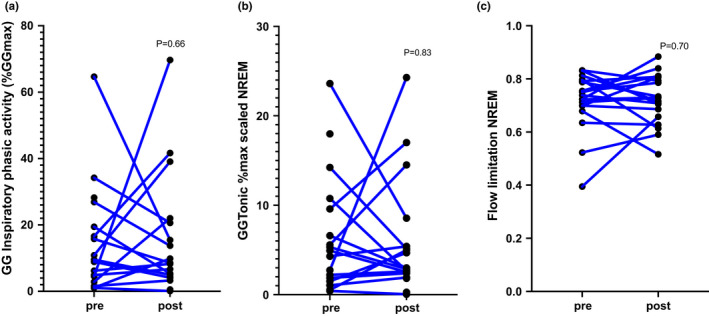
(a) GG‐peak phasic inspiratory activity, (b) GG‐tonic, (c) flow:Drive NREM did not significantly change following intervention.

Snoring time during the PSG did not significantly change (Table 1 and Figure 2c). Model estimates of flow limitation severity (flow:drive) did not reveal any significant change from baseline to follow up (Table 1 and Figure 2d). Findings were similar using mixed modeling.

### 3.2 Secondary outcomes

Polysomnographic measures showed a significant increase in sleep efficiency, but not in the AHI, nadir oxygen saturation, or arousal index (Table 1).

There was a significant improvement in PSQI (5.7 ± 0.59 vs. 4.9 ± 0.47; *p* = 0.03). Median ESS was unchanged pre‐intervention and post‐intervention (5.5 ± 0.50 vs. 5.2 ± 0.40; *p* = 0.37).

Psychomotor vigilance testing showed an improvement in number of false starts, but not in reaction time or number of lapses >500 ms (supplemental table). Supplemental data provided at: https://figshare.com/articles/figure/supplemental_pptx/14879730.

**FIGURE 3:**
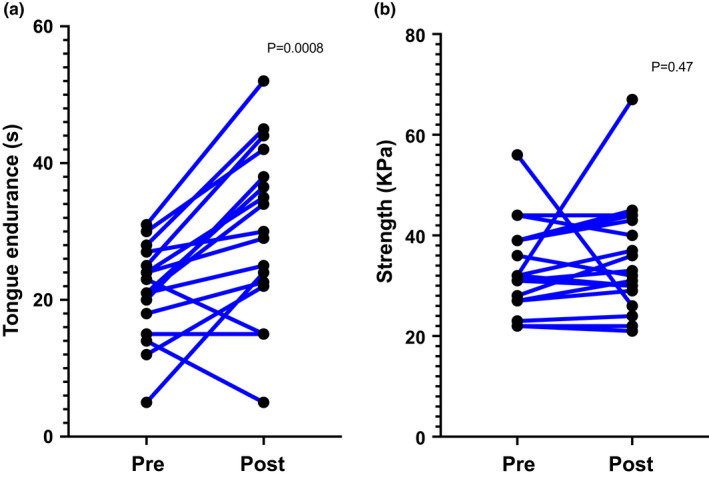
(a) Tongue endurance data as assessed by Iowa intraoral pressure instrument (IOPI) using time spent above 50% max strength (seconds). (b) Tongue strength data as assessed by Iowa intraoral pressure instrument (IOPI) as measured in kilopascals (KPa). One subject was omitted for not following IOPI instructions.

Data from intraoral pressure assessments showed a significant increase in tongue endurance (time spent at greater than 50% max strength; 21.7 ± 1.6 vs. 37.0 ± 3.0 s; *p* = 0.03), but not in strength (33.2 ± 1.9 vs. 34.5 ± 2.4 kPa; *p* = 0.59; Figure 3).

## 4 DISCUSSION

We did not detect a major impact of NMES on genioglossus EMG activity during sleep. Given that we did not observe a decrease reported snoring or flow limitation, the lack of change in genioglossus activity may be expected in that inspiratory flow limitation is a major contributor to genioglossal activation. However, we did see changes following NMES that may indicate a treatment effect, including increased tongue endurance associated with improved PSQI, and improved PVT, even in those with mild baseline flow limited breathing. In addition, the therapy was generally well tolerated with no subjects dropping out due to side effects or intolerance. The two patients who did drop out did not use the device on a routine basis.

Of the potential mechanisms for flow limitation assessed, neural drive to the genioglossus does not appear to be effected by NMES. Importantly however, there are multiple mechanisms through which flow‐limited breathing can arise, such as: intrinsic anatomic collapsibility of the upper airway, decreased neural drive to pharyngeal dilators, insufficient pharyngeal dilator strength (despite increased neural drive to genioglossus in OSA), as well as negative effort dependence where pharyngeal collapse evolves in proportion to respiratory efforts (Schmickl et al., in press; Saboisky et al., 2007; Saboisky et al., 2012; Owens et al., 1985). Only neural drive and measurements of tongue strength/endurance we assessed in the present study. Notably, we did find some improvement in awake assessment of tongue endurance with NMES training. This increase in tongue endurance may be important for ensuring airway patency in the context of repetitive obstructive events.

Accordingly, there are several areas that need investigation to optimize this therapy: First, for stimulation/training during wakefulness, the “dose” needed for optimal training is not clear. Adequate stimulation is clearly important, but excessive stimulation might cause muscle injury and therapy intolerance. Second, selection of individuals likely to respond to upper airway muscle training is needed given the spectrum of underlying anatomic and non‐anatomic OSA traits (i.e., “endotypes”) seen across individuals; those with impaired upper airway dilator function alongside more mild upper airway collapsibility might be ideal. Markers of individuals in whom enhancement of tongue activity will alleviate upper airway obstruction would be applicable here as well as with hypoglossal nerve stimulation (Cori et al., 2018).

Tongue activation has a complex relationship with inspiratory flow limitation during spontaneous breathing, which creates a challenge for mechanistic investigation and selection of potential NMES responders. While we do not know whether genioglossus is explicitly activated by the NMES device, it is a reasonable candidate given the position of the stimulation probes. Additionally, genioglossus is both necessary and sufficient for the maintenance of pharyngeal patency during sleep (Stanchina et al., 2002). Notably, increasing genioglossus activity typically leads to decreased flow limitation, but decreased flow limitation subsequently may reduce genioglossus activity. Investigators have used techniques to manipulate upper airway collapse in order to control these factors, whereby one can determine the electromechanical effectiveness of genioglossus by examining whether increased activity leads to decreased flow limitation. However, given the mildness of disease, these techniques were not pursued in the current set of experiments.

These traditional methods to determine OSA endotypes may be used to predict which individuals may benefit from non‐PAP therapy (Owens et al., 2015). More recently, modeling techniques have facilitated measurement of pharyngeal collapse as it relates to estimated ventilatory drive (Sands et al., 2018a). This method has already shown promise towards predicting responses to oral appliances and oxygen (Sands et al., 2018b; Vena et al., 2020). Validating such techniques to determine the effectiveness of genioglossal activation will help to understand mechanisms and individualize treatments.

At follow up, we observed an improvement in sleep quality as assessed by PSQI and some improvement in sleep efficiency as measured by polysomnography, though it is unclear to what extent improvements in PSQI are clinically significant (Buysse et al., 2008). We note that these findings could have been related to the “first night effect” although should reflect the 4‐week pre‐treatment vs 4 week on‐treatment period. Similarly, we observed some improvement in psychomotor vigilance parameters which can have a learning effect (Basner et al., 2018). However, it is established that snoring and mild OSA have the potential to impact sleep quality (Arnardottir et al., 2016). One potential explanation for the observed improvements in PSQI, sleep efficiency, and PVT following neurostimulation is that subtle improvements in breathing and sleep are not captured by our measures of snoring and flow limitation.

### 4.1 Limitations

Despite our study's strength and novelty, we acknowledge many limitations. First, given the invasive nature of our physiological assessments, our sample size was modest. Thus, we are clearly underpowered for small effect sizes or subgroup analyses. Although we did not see systematic differences in genioglossus activity between groups, we acknowledge that these recordings are highly variable and thus we may require a large sample to see statistical significance or to identify which subsets respond best to therapy. Future trials should be directed towards clinical outcomes rather than surrogate metrics. In addition, some individuals who passed screening had higher AHIs than intended based on inclusion criteria on PSG, and thus our population was likely more heterogenous than intended. Second, the rigorous measurement of snoring is notoriously difficulty since self‐report, bed partner report, objective measurements etc. all give slightly different information. We used both snoring as well as objective quantification of inspiratory flow limitation to attempt to examine any effect but arguably none are definitive. Thus, we encourage further efforts in this context. Third, despite our best efforts, treatment adherence was not 100%, which may have decreased the impact of our intervention. We view this finding as the nature of human research rather than a major deficiency in our approach or in the technology we were assessing. Notably, we view it as a limitation that this model of the device did not allow for correlation of device intensity and clinical outcomes. Newer versions of the device have cloud‐based monitoring with adherence and intensity data routinely available. Lastly, our study did not have a sham control group, so we cannot rule out changes based on time or repeat testing, as noted above. Despite these limitations, we view our findings as important and hope that they lead to further studies in this area.

## 5 CONCLUSIONS

Our study assessed the physiological effects of transoral neurostimulation on genioglossus activity and markers of inspiratory flow limitation in individuals with snoring and mild OSA, but an effect on these particular parameters was not observed. We saw positive results towards increased tongue endurance, improved PSQI, and improved PVT, even in those with mild baseline flow limited breathing. Further study is needed across a broader cohort in order to assess the ideal patient population for this device.

### Funding and disclosures

This study was funded by Signifier medical. The authors did not receive any compensation for completing this study. The following is a list of general funding sources: Dr. Nokes is supported by the NIH [T32 grant HL134632], Sleep Research Society Career Development Award, as well as the American Thoracic Society ASPIRE Fellowship. Dr. Schmickl received salary support from NHLBI (T32 grant HL134632 “Training the Next Generation in Respiratory Science”) and the ATS Foundation during the conduct of the study.

## Disclosures

Nokes, Schmickl, Bhattacharjee andMann have nothing to disclose. Dr. Sands reports income from Nox Medical (consulting), Merck (consulting), Inspire (consulting), Apnimed (grant support), Prosomnus (grant support).Owens is funded by the NHLBI (HL142114), and reports personal fees from Novartis and Nitto Denko Asia, outside the submitted work.Malhotra is funded by NHLBI. He received income from Livanova, Corvus and Equillium for medical education. Orr receives research funding from NHLBI, and advisory board compensation from ResMed. ResMed provided a philanthropic donation to UC San Diego.

## AUTHOR CONTRIBUTIONS

Brandon Nokes, Christopher N. Schmickl, Robert L. Owens, Atul Malhotra, and Jeremy E. Orr designed and executed the study, performed data analysis, wrote and edited the manuscript. Rebbecca Brena, Nana Naa‐Oye Bosompra, Dillon Gilbertson, and Rakesh Bhattacharjee aided in experimental completion and editing the primary manuscript. Dwayne L. Mann, Scott A. Sandsaided in data analysis writing/revising the manuscript.

## Supporting information




**Appendix S1** Supporting Information

## Data Availability

Data available on reasonable request from the authors.
