# Supplementary material for: The impact of daytime transoral neuromuscular stimulation on upper airway physiology – A mechanistic clinical investigation
Source: Physiol Rep. 2022 Jun 24;10(12):e15360. doi: 10.14814/phy2.15360 (PMC9226850; doi:10.14814/phy2.15360)
Supplement: Supplementary file 1 — Appendix S1 Supporting Information [file PHY2-10-e15360-s001.pptx]

## Slide 1
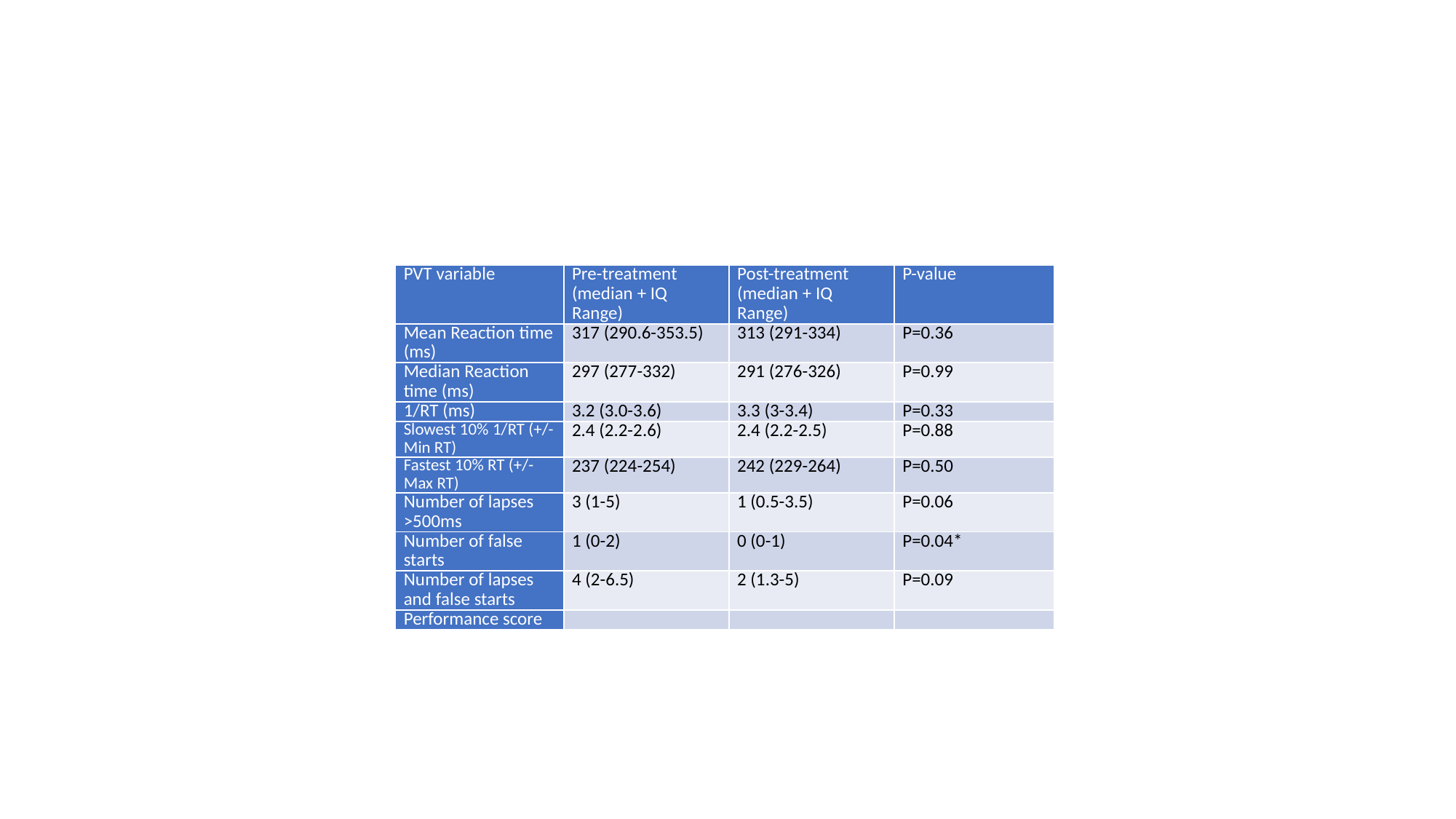

| PVT variable | Pre-treatment (median + IQ Range) | Post-treatment (median + IQ Range) | P-value |
| --- | --- | --- | --- |
| Mean Reaction time (ms) | 317 (290.6-353.5) | 313 (291-334) | P=0.36 |
| Median Reaction time (ms) | 297 (277-332) | 291 (276-326) | P=0.99 |
| 1/RT (ms) | 3.2 (3.0-3.6) | 3.3 (3-3.4) | P=0.33 |
| Slowest 10% 1/RT (+/- Min RT) | 2.4 (2.2-2.6) | 2.4 (2.2-2.5) | P=0.88 |
| Fastest 10% RT (+/- Max RT) | 237 (224-254) | 242 (229-264) | P=0.50 |
| Number of lapses >500ms | 3 (1-5) | 1 (0.5-3.5) | P=0.06 |
| Number of false starts | 1 (0-2) | 0 (0-1) | P=0.04\* |
| Number of lapses and false starts | 4 (2-6.5) | 2 (1.3-5) | P=0.09 |
| Performance score | | | |

## Slide 2
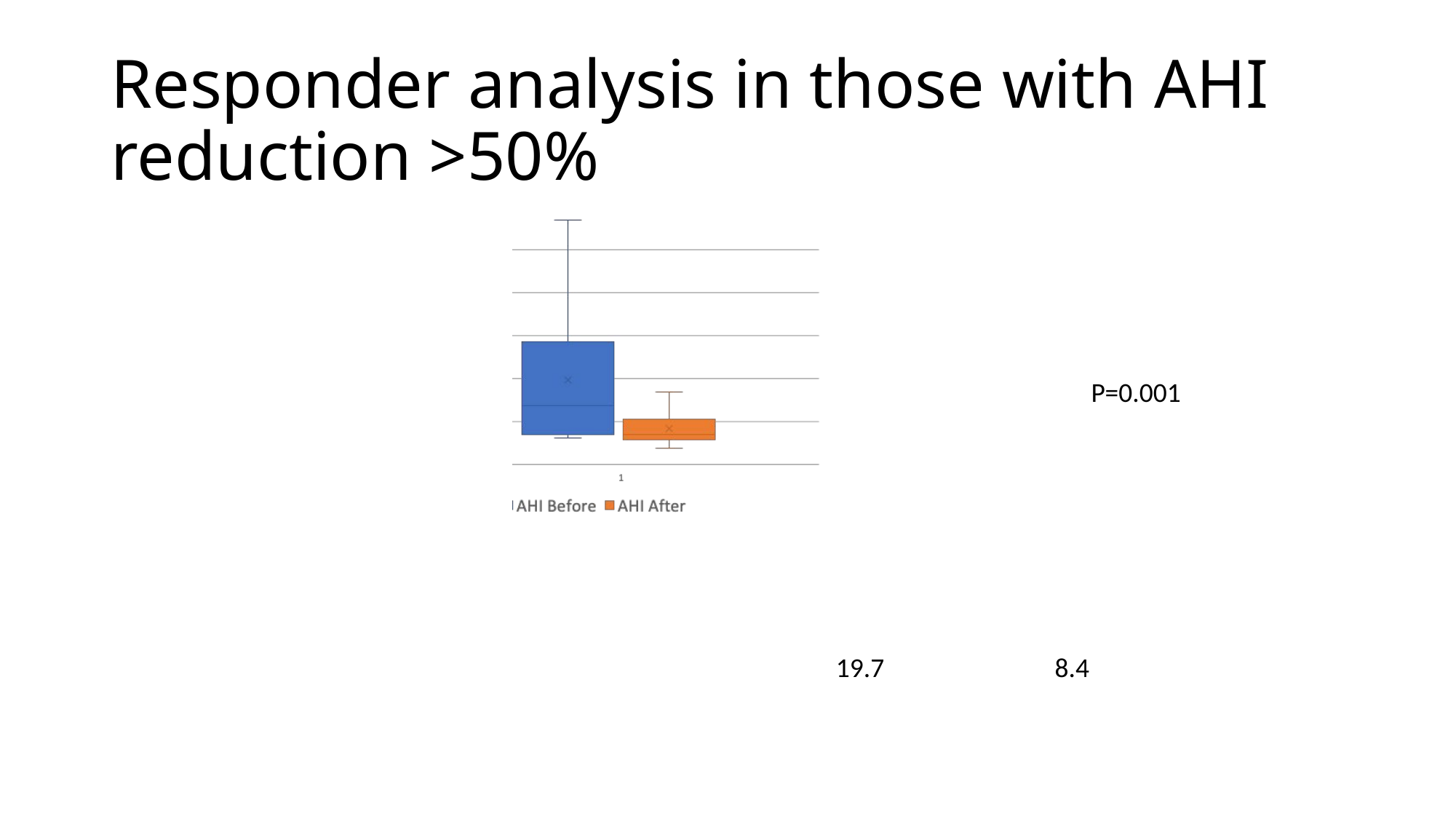

# Responder analysis in those with AHI reduction >50%
19.7
8.4
P=0.001
